# The Evolution of China’s Railway Network (CRN) 1999-2019: Urbanization Impact and Regional Connectivity

**DOI:** 10.1007/s40864-022-00168-9

**Published:** 2022-06-23

**Authors:** Wei Wang, Wenbo Du, Kun Liu, Lu Tong

**Affiliations:** 1 School of Electronic and Information Engineering, Beihang University, Beijing, 100191 China; 2 HCIG Communication Investment Co., Ltd, Shijiazhuang, 050051 China; 3 Research Institute of Frontier Science, Beihang University, Beijing, 100191 China

**Keywords:** China’s Railway Network, Complex network, Network topology, Train flow, Travel distance, Regional connectivity

## Abstract

With the rapid development of China’s economy and society, China’s railway transportation system has been dramatically improved in terms of its scale and operational efficiency. To uncover the underlying relationship between urbanization and railway network structure, this paper examines the evolution of China’s railway transportation system from 1999 to 2019 by applying complex network theory. The results show that China’s railway network (CRN) has become more connected, more “small-world” and more heterogeneous since the beginning of the twenty-first century. Based on the train flow and train travel distance, the evolutionary course of CRN is found to undergo two apparent stages, with a turning point in 2007. By calculating the regional railway connection index (RRCI), it is revealed that the planned core cities in different regions act as bridges connecting the regions to the rest of the whole network.

## Introduction

Railways, characterized by high capacity, solid travel time reliability, relatively cheap fares, and low carbon emissions, are closely associated with a country’s socioeconomic development and have received considerable attention from scientists in statistics [1], geography [2], transportation [3], and other research fields. As a system of complicated connections of hundreds of cities, the structure of the railway network is affected by a variety of factors, such as political policy, regional population, and geographic conditions, and therefore is commonly studied as a typical complex system. In recent years, the widely used complex network theory [4–6] has developed into a powerful tool to analyze the network performance for different transportation modes, including aviation [7–9], roads [10, 11], and shipping [12–14], and also provides a new perspective for understanding how railway transportation systems work. A series of related studies have investigated topological structure, traffic behavior, and cascading failure on continental [15], national [16, 17], and urban scales [18, 19], respectively. Sen et al. studied the structural properties of the Indian railway network (IRN) and showed that the IRN is endowed with small-world property [20]. Further, Ghosh et al. found that the traffic flow of the railway network is related to its topological characteristics. They investigated the IRN as a weighted complex network and revealed that traffic is accumulated in stations with high connectivity and the links between them [21]. Various literature has emerged relating to the analysis of China’s railway network (CRN). Li and Cai studied the CRN [22] and showed that in addition to a common small-world feature, CRN is characterized by other striking structural properties including scale-free distributions, heterogeneous connectivity, and hierarchical modularity. These properties may give rise to particular network vulnerability when important nodes are destroyed [23]. There are also studies focusing on urban railway networks. To understand their complex characteristics, various network measures have been developed. Proposing three network reliability indicators, Liu et al. [24] found that Wuhan’s subway network is susceptible to the failure of important stations, with the geographically central stations playing a significant role in maintaining network reliability. To [25] introduced five centrality measures to conduct network analysis of Hong Kong’s urban rail system and reported that betweenness centrality showed superior performance in reflecting the transport loadings of a rail station. To further distinguish the difference in rail line technology, Sharav et al. [26] extended the basic network measures by adding weights to compare different transit modes. Focusing on the newly built metro networks, Zhao et al. [27] examined the relationships among statistical parameters such as travel cost, chessboard coefficient, and vulnerability in second-tier cities.

Evolutionary analysis of transportation systems has also been a popular research direction [28, 29]. As the expansion of railways plays an important role in shaping the transportation structures of countries, regions, and cities, the dynamic evolution process of railway networks has drawn increasing attention. Marti-Henneberg [30] analyzed the evolution of railways in Europe from 1840 to 2010 on the basis of the railway lines in service and the changes in state economic geography. It was revealed that every national railway network has exhibited unique characteristics even though they have similar guidelines. Murayama [31] measured the time-based connectivity of the Japanese railway network (JRN) using a time-distance matrix to explore how the nationwide expansion of the JRN achieved a time-shrinking effect during 1868-1990 and showed that travel time was reduced by 80% over the course of the century. Moreover, they pointed out that the cities connected by high-speed lines had the most significant gains. The evolution of CRN has also been widely studied as China has experienced a period of rapid CRN development in recent decades. Wang et al. [32] examined the expansion of CRN in the twentieth century and revealed an evolutionary process from “preliminary construction” to “deep intensification”. Further, Jiao et al. [33] adopted three centrality indicators with regard to network topology to examine the changes in node connectivity in China’s high-speed rail network from 2003 to 2014 and showed that network connectivity was significantly improved. Xu et al. [34] proposed a connectivity-accessibility index to assess the impact of the future railway network structure on the potential development of cities. The results show that cities in the Yangtze River Delta would suffer the most, whereas cities in the central and western regions would gain the most.

Although progress has been made in investigating the evolution of CRN, previous studies are mostly concentrated on the evolution law of the network structure. It is generally known the many parts of China differ in geographic settings, economic bases, and population [35]. CRN establishes connections among cities and thus presents specific attributes in economic geography. Studying the complex coupling relationship would be helpful to further explore the major influence of unique demographic and geographical attributes and reveal the underlying mechanism of CRN evolution in detail. In this context, we aim to investigate the evolutive characteristics of the entire CRN by combining the structural topology with the traffic dynamics during the period from 1999 to 2019. The development of high-speed trains allows for stronger spatial interaction of cities and redistribution of economic activities within regions. Finally, we explore the evolution of connections at regional levels and analyze the similarities and differences among different regions.

In summary, a number of scholars have applied complex network theory to explore the structure and dynamics of different transportation networks. Existing literature related to railway networks mainly focuses on characteristics of static networks. Little attention has been paid to the evolution of regional railway internal/external interactions over the long term. Table 1 shows a comparison between our work and existing literature. The major contributions of this paper are as follows: (1) We explore the underlying relationship between urbanization and railway network structure by integrated analysis of network topology and train flows. (2) A regional railway connection index is proposed to uncover regional evolution processes of CRN. We hope that our work will contribute to a better understanding of CRN and provide a reference for evolution analyses of other transportation networks.

**Table 1:** Comparison between our work and existing literatures

Publications	Network types	Time span	Research content			
			Transportation mode	Traffic flow	Urbanization impact	Regional connectivity
Calzada-Infante et al. (2020) [15]	Static	2018	European railway network	Train frequency	City	–
Wang et al. (2020) [16]	Static	2016	China’s railway network	Number of trains	City, region	Intra-region/inter-region time-based accessibility index
Cao et al. (2019) [17]	Static	2017	China’s high-speed railway network	Train frequency	City	–
Sen et al. (2003) [20]	Static	–	Indian railway network	–	–	–
Ghosh et al. (2011) [21]	Static	2010	Indian railway network	Number of trains	City, region	–
Li et al. (2007) [22]	Static	–	China’s railway network	Number of trains	–	–
Ouyang et al. (2014) [23]	Static	–	China’s railway network	Number of trains	–	–
Latora et al. (2002) [18]	Static	–	Boston subway network	–	–	–
Xu et al. (2016) [36]	Static	2014	Beijing urban rail network	Number of passengers	City	–
Li et al. (2019) [19]	Static	2016	Beijing urban rail network	Number of passengers	–	–
Tian et al. (2018) [37]	Static	–	Urban bus network	–	City	–
Yue et al. (2019) [38]	Static	2017	Railway-coach coupled network	Number of passengers	City, region	Intra-region/inter-region distance-based proximity index
Feng et al. (2021) [39]	Static	2020	Railway-airline coupled network	Number of trains/flights	City, region	–
Zhang et al. (2010) [28]	Dynamic	1950-2008	China’s airline network	Number of passengers/cargos	City	–
Gallotti et al. (2015) [29]	Dynamic	18-24 October 2010	UK public transport network	–	–	–
Wang et al. (2009) [32]	Dynamic	1906-2000	China’s railway network	Number of passengers/cargos	City, region	–
Jiao et al. (2017) [33]	Dynamic	2003-2014	China’s high-speed railway network	Train frequency	City	–
Xu et al. (2018) [34]	Dynamic	2007-2030	China’s high-speed railway network	–	City, region	–
Huang et al. (2018) [40]	Dynamic	2008-2017	China’s high-speed railway network	Number of trains	City	–
Feng et al. (2019) [41]	Dynamic	12-18 December 2016	Paris subway network	Number of trains	–	–
Our work	Dynamic	1999-2019	China’s railway network	Number of passengers/cargos, number of trains	City, region	Intra-region/inter-region traffic-based connection index

The rest of this paper is organized as follows. In Sect. 2, a statistical description of the railway transportation industry is presented. A national-scale evolution analysis of CRN including topological properties, train flows, and geographical properties is given in Sect. 3. Section 4 examines the regional evolution characteristics of CRN, and a conclusion is drawn in Sect. 5.

## Overview of China’s Railway Development

**Fig. 1:**
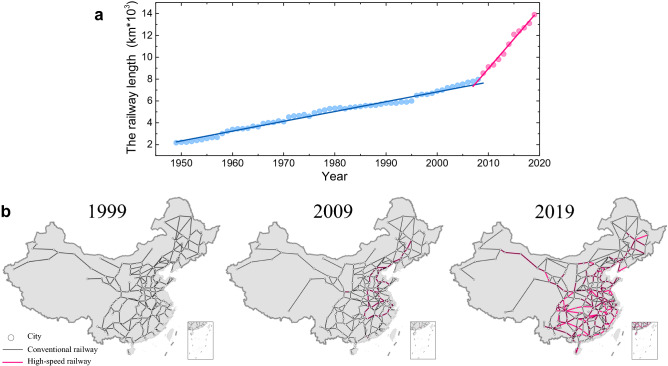
The overview of China’s railway construction. **a** The railway length from 1949 to 2019. **b** The spatial distribution of railway lines in 1999, 2009, and 2019. Data sources: National Bureau of Statistics and Schematic Representation of National Railway Lines

Over the past few decades, China’s railway system has made great strides. Figure 1a shows the expansion of China’s railway system over the period 1949-2019, with two distinct phases. Figure 1b shows the spatial distributions of railway lines in 1999, 2009, and 2019. In 1999, the total railway operation length reached 67,400 km [42], which was approximately twice that in 1949, and ranked first in Asia and fourth in the world [42]. As of 2009, the total length of railway lines exceeded 85,000 km [43], ranking second in the world [43]. At the same time, with an increasing emphasis on the planned construction of high-speed rail (HSR), China started to build HSR lines in the eastern area, and over the following 10 years shaped the national HSR network comprising eight vertical (north-south) and eight horizontal (west-east) corridors. In 2019, although the number of cities linked by conventional railway was not significantly increased, more cities were covered by the HSR with efficient transportation services.

**Fig. 2:**
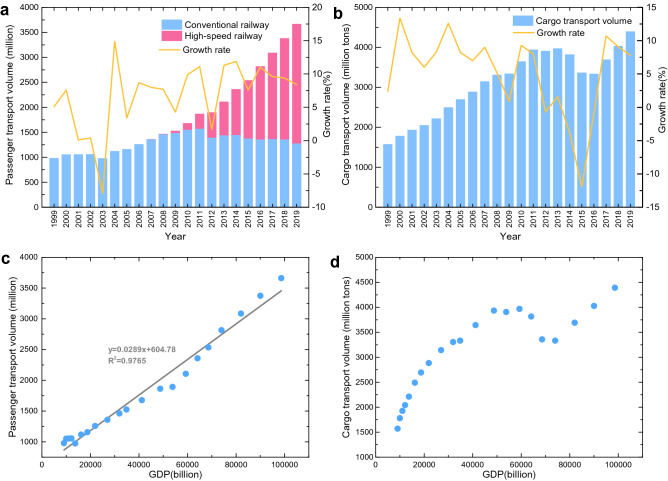
China’s railway passenger and cargo traffic and their relation with GDP. **a** Passenger transport volume. **b** Cargo transport volume. **c** Relation between passenger transport volume and GDP. **d** Relation between cargo transport volume and GDP. Data sources: China Railway Statistical Yearbook, 1999-2019

From a macroeconomic perspective, we analyze the changing railway traffic (passengers and cargos) and their association with economic growth [44]. In Fig. 2, it is observed that passenger transport volume has generally grown year by year (an average annual increase of 6.83%). In particular, it experienced more rapid growth from 2009 onward (an average annual increase of 9.15%), which is mainly attributed to the HSR (marked by red). The HSR has become a major transportation mode for medium/long-distance passengers, carrying over 60% of total railway passengers in 2019. However, passenger transport volume of the conventional railway (marked by blue) dropped slightly after 2009. Unlike railway passengers, the total railway cargo volume did not show a consistent rise. To be more specific, the growth of cargos stagnated in 2012, dropped significantly in 2015, and started to recover again in 2017. Due to the continuous economic structure adjustment and energy structure optimization, large-quantity cargo traffic (coal, mineral, steel, etc.) initially dropped from 2012 and then rose in the following years. It is also worth mentioning that passenger traffic was greatly affected (7.9% decrease) by the outbreak of severe acute respiratory syndrome (SARS), an infectious disease, in 2003. However, railway cargo traffic appeared unaffected in the same year. Additionally, as shown in Fig. 2c, d, passenger traffic grew almost linearly with gross domestic product (GDP), while cargo traffic did not show a similar tendency.

## Evolutive Properties of CRN

### Construction of CRN

**Fig. 3:**
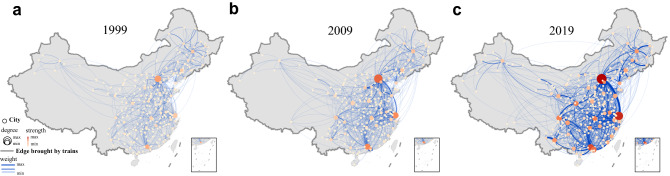
The representation of CRN. The network in **a** 1999, **b** 2009, and **c** 2019. Here, nodes represent cities (multiple stations in the same city are represented by a single node) and edges stand for train schedules between cities. Node size is positively correlated with node degree (i.e., the number of connections) and node color reflects node strength (i.e., the number of departing or arriving trains). Both thickness and color of edges represent the weight of edges (i.e., train flow)

Typically, the definition of railway network depends on the focused questions in research. Network models can be divided into two main categories: physical networks (e.g., Space K [32]) and logical networks (e.g., Space P [20], Space L [22], and Space G [45]). In physical networks, nodes and edges represent real entities in the topological structure, while in logical networks, the topological structures are formed in accordance with some artificially defined rules, and the network elements are partially or totally virtual. We collected railway physical infrastructure data and passenger train schedules for 20 years (1999-2019) to examine the evolution of China’s railway transportation system from the perspective of the logical network. The data set involves 3187 stations and 81,081 train schedules in total in mainland China. In this study, nodes are defined as cities. Multiple stations in the same city have been merged into one node. Two nodes are connected by an edge if they are the starting and ending cities of a train schedule, respectively. The geographical representation of CRN in 1999, 2009, and 2019 is shown in Fig. 3. Obviously, CRN has a progressive tendency to form a denser network with high-traffic links.

### Topological Properties of CRN

This section discusses the evolution of the topological properties of CRN from 1999 to 2019 (see Table 2). In general, CRN increased significantly in that time period despite some fluctuations. Specifically, the number of nodes increased by 21.68% from 226 in 1999 to 275 in 2019; edges increased by 83.72% from 682 in 1999 to 1253 in 2019. The average degree <*k*> continued to grow, which means that CRN was more closely connected in the same period. The increase in clustering coefficient *C* and the reduction in average shortest path length *d* indicate more prominent small-world properties. Furthermore, the decreased assortativity coefficient *r* with a negative value implies CRN’s higher disassortative level and more heterogeneous network structure. Based on measurements of these network topology indices, CRN was more densely reticulated, more “small-world”, and more heterogeneous across that period.

**Table 2:** Evolution of topological parameters of CRN from 1999 to 2019

Year	*N*	*E*	<*k*>	*C*	*d*	*D*	*r*
1999	226	682	6.04	0.329	3.04	7	−0.062
2001	216	717	6.64	0.398	2.91	6	−0.089
2003	212	709	6.68	0.375	2.91	6	−0.104
2005	212	716	6.75	0.387	2.85	6	−0.121
2007	214	738	6.90	0.389	2.78	6	−0.173
2009	229	837	7.31	0.400	2.79	7	−0.17
2011	236	854	7.24	0.426	2.77	7	−0.175
2013	234	886	7.57	0.419	2.77	6	−0.179
2015	252	1027	8.15	0.480	2.71	6	−0.177
2017	271	1193	8.80	0.508	2.63	6	−0.205
2019	275	1253	9.11	0.516	2.62	5	−0.204

*N* represents the number of nodes.* E* represents the number of edges. *<k>* means an average degree. *C* is the overall level of the clustering coefficient in the network. *d* is the average shortest path length of the entire network, and *D* is the diameter.* r* is the assortativity coefficient ranging between -1 and 1

**Fig. 4:**
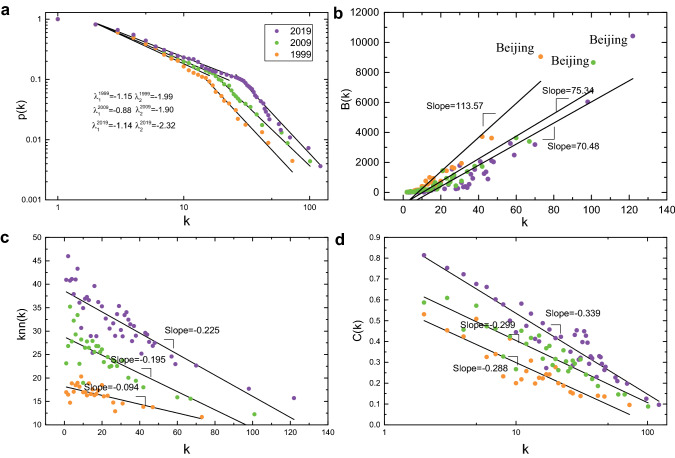
Topology indices of CRN (1999-2019). **a** The cumulative degree distribution *p*(*k*); **b** *B* (betweenness) versus *k* (degree); **c** *k*_*nn*_ (degree correlation) versus *k* (degree); **d** *C* (clustering coefficient) versus *k* (degree)

The network topology in 1999, 2009, and 2019 is further evaluated in Fig. 4. Cumulative degree distribution, one of the most important properties, is measured and shown in Fig. 4a. It follows a two-regime power law during the 3 years, indicating a common phenomenon, namely an inhomogeneous connectivity distribution, where a minor fraction of nodes hold many connections, while most nodes have only sparse connections [46]. Additionally, the “two-regime” inflection points are progressively right-shifted (${k}_{1999}=15$, ${k}_{2009}=20$, ${k}_{2019}=26$). Interestingly, cities with a degree larger than that of the inflection points are usually municipalities, provincial capitals, and sub-provincial cities. In particular, Shenzhen was included in the high-degree group in 2009, and Fuzhou, Xiamen, and Ningbo were included in 2019. Betweenness, a popular topological index, is widely used to quantify node transitivity in an overall network. The calculation of betweenness is equal to the fraction of all shortest paths passing through a given node. Figure 4b shows the relation between degree and betweenness in 1999, 2009, and 2019. We further observe that the slope gradually decreased over time. With the additional railway hubs, the gap of transfer load between different high-degree nodes further narrowed. It is worth noting that Beijing, the capital city of China, consistently deviated from the fitting lines. The degree correlation (the mean degree of neighbors of a node) and clustering coefficient (the proportion of interconnected neighbors of a node) as functions of the degree are illustrated in Fig. 4c, d, respectively. It is evident that these two correlations are inversely related to the degree, and the slope of fitted lines gradually decreases with time. In each individual year, the smaller the degree of one node, the larger the average degree of its neighbor nodes and the more dense the connection between these neighbors. The hierarchical structure of CRN became more pronounced year by year [47].

### Evolution of the Train Flow and Train Travel Distance

**Fig. 5:**
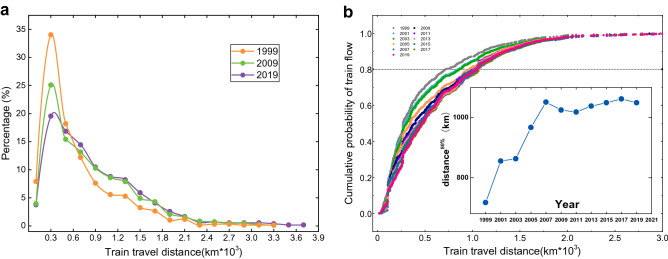
The impacts of the topological evolution of CRN (1999-2019) on the train flow and train travel distance. **a** The distribution of train travel distance; **b** cumulative probability of train flow over train travel distance. The evolution of train travel distance under 80% of the total train flow is shown in the inset

The train flow between cities is always constrained by travel distance. Technological innovations in the rail sector have brought a shrinkage of space and influenced spatial interactions among cities [33]. For a deeper understanding of the functional properties of CRN, the relationship between train flow and train travel distance is analyzed. As Fig. 5a shows, the edges with train travel distances within 300 km accounted for 41.92% in 1999. Nevertheless, the percentage decreased by 12% in 2009 and continued to decline by 5.7% in 2019. This suggests that a larger portion of trains performed long-distance transport tasks owing to high-speed rail technology. Figure 5b exhibits the evolution of the cumulative probability of train flow over train travel distance from 1999 to 2019. The thresholds of distances with 80% of the train flows are extracted and shown in the inset. From 1999 to 2007, these distance thresholds increased significantly, whereas they remained relatively stable after 2007, when high-speed trains started to be put into service and became a primary way to satisfy the growing transportation demand. One interesting point is that high-speed trains did not continue to facilitate the establishment of more new connections among long-distance nodes, instead they mainly increased the train flow of existing connections.

**Fig. 6:**
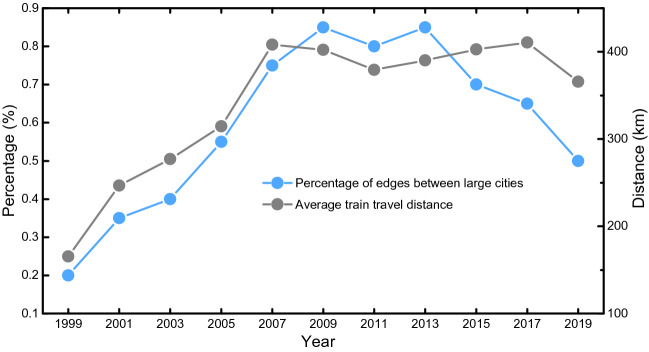
The percentage of edges between large cities and the average train travel distance of the top 20 edges

The train flow between different city pairs is further studied to reveal the year-by-year evolution of CRN. Table 3 tabulates the top 20 edges with the highest train flow in 1999, 2007, and 2019 (here the year 2007 is selected instead of 2009 since an inflection point appeared in 2007 in Fig. 5b). Municipalities, provincial capitals, and sub-provincial cities are referred to as large cities, and the rest as small cities. More specifically, it can be seen that the edges of Beijing–Tianjin and Guangzhou–Shenzhen consistently appeared and remained the top three during the 3 years. These two edges are respectively distributed in the most developed regions and have a short railway travel distance. In 1999, 70% of edges were completely or partially connected to large cities. The remaining edges spanned almost the whole of northeastern China, due to the relatively dense network formed during the “Northeastern Region Period” [32]. In 2007, more edges between larger cities were added to the list of the top 20 edges. The average train travel distance of the top 20 edges reached about 408.16 km, which was twice as long as that in 1999. In 2019, although train speed continued to increase, long-haul edges connecting large cities did not maintain a high ranking. It was found that large cities had stronger interactions with their nearby satellite cities, and the average train travel distance of top 20 edges declined compared to 2007. It has been reported that high-speed rail acts as a catalyst in facilitating region integration [48]. This may explain why Changsha–Xiangtan, Changsha–Zhuzhou, and Zhengzhou–Jiaozuo were added to the top 20 list. Figure 6 illustrates the percentage of large city pairs (denoted by the blue line) and the percentage of average train travel distance (denoted by the gray line) of the top 20 edges year by year. In general, the proportion of large city pairs first rose and then declined. We also found that the average train travel distance initially shows an obvious increase before 2007 with the increase in long-haul edges between larger city pairs, and subsequently tends to be relatively stable.

**Table 3:** Top 20 edges ranked by train flow in 1999, 2007, and 2019

	1999		2007			2019		
	Edge	Distance (km)	Edge	Distance (km)	Time saved (h)	Edge	Distance (km)	Time saved (h)
1	**Guangzhou–Shenzhen**	111.68	**Guangzhou–Shenzhen**	111.68	0.22	**Beijing–Tianjin**	108.64	0.60
2	**Beijing–Tianjin**	108.64	**Shanghai–Nanjing**	268.12	0.83	**Guangzhou–Shenzhen**	111.68	0.38
3	**Shenyang**–Fushun	42.84	**Beijing–Tianjin**	108.64	0.08	**Shanghai–Nanjing**	268.12	0.78
4	**Beijing–Shijiazhuang**	269.29	**Shanghai–Hangzhou**	165.24	0.60	**Beijing–Shanghai**	1063.80	5.68
5	**Harbin**–Qiqihar	267.29	**Beijing–Shijiazhuang**	269.29	0.72	**Kunming**–Dali	256.52	5.40
6	**Harbin**–Suihua	102.54	**Beijing**–Qinhuangdao	269.90	2.77	**Changchun**–Jilin	100.58	0.92
7	**Harbin**–Mudanjiang	267.36	**Jinan–Qingdao**	304.97	1.58	**Changsha**–Xiangtan	36.75	1.35
8	**Shanghai–Hangzhou**	165.24	**Beijing–Shanghai**	1063.80	4.02	**Shanghai–Wuhan**	674.29	1.85
9	**Nanchang**–Jiujiang	116.19	**Shanghai–Ningbo**	154.57	3.15	**Shenyang–Dalian**	352.55	1.83
10	Qiqihar–Hulun Buir	378.22	**Beijing–Qingdao**	545.94	8.40	**Harbin**–Jiamusi	310.66	4.05
11	**Beijing**–Zhangjiakou	161.31	**Beijing–Wuhan**	1049.57	3.77	**Changsha**–Zhuzhou	42.24	0.63
12	**Harbin**–Jilin	211.56	**Chengdu–Chongqing**	269.93	6.88	**Zhengzhou**–Jiaozuo	65.53	1.77
13	Hegang–Jiamusi	60.05	**Shenyang–Dalian**	352.55	0.72	**Xi’an–Chengdu**	607.12	12.15
14	Heihe–Qiqihar	411.53	**Beijing**–Handan	404.54	1.22	**Guangzhou**–Zhanjiang	367.20	3.57
15	Jixi–Mudanjiang	133.63	**Beijing–Shenyang**	622.24	5.52	**Chengdu–Chongqing**	269.93	2.27
16	**Changchun**–Jilin	100.58	**Harbin**–Qiqihar	267.30	0.25	**Shanghai**–Wenzhou	371.13	5.90
17	Jiamusi–Shuangyashan	61.14	**Beijing–Zhenzhou**	623.41	1.98	**Shenyang**–Dandong	203.71	2.38
18	Lianyungang–Xuzhou	201.84	Lianyungang–Xuzhou	201.84	1.07	**Changchun**–Yanbian	355.71	5.25
19	**Xi’an**–Weinan	53.89	**Guangzhou–Chongqing**	976.06	22.87	**Guangzhou–Wuhan**	837.29	9.93
20	**Shenyang**–Anshan	83.08	Jixi–Mudanjiang	133.63	0.32	**Beijing–Xi’an**	910.25	6.70
	Average	165.40	Average	408.16	3.35	Average	365.69	3.67

Here, edges that appear in the top 20 list in all 3 years are underlined. The municipalities, provincial capitals, and sub-provincial cities are in bold

## Regional Evolution Characteristics of CRN

The evolution of CRN has not only triggered changes in overall railway structure, but has also promoted internal and external connection of regions. We selected 19 regions (i.e., megalopolis, urban agglomeration, or city groups) proposed in the 13th Five-Year Plan [49] as our areas of interest. To evaluate the role a city plays in the regional railway passenger transport, we develop a regional railway connection index (RRCI) ${R}_{i}$ for city $i$ as the proportion of inside-region train flow ${S}_{i{-}{in}}$ to outside-region train flow ${S}_{i{-}{out}}$ as follows.

1 $${R}_{i}=({S}_{{i}{-in}}+1)/({S}_{{i}{-out}}+1)$$

When ${R}_{i}>1$, it indicates that city $i$ has an advantage in connecting cities within the region; when ${R}_{i}<1$, it means this city fosters more interactions with cities outside the region. We define a city as a regional internal advantage (RIA) city if its RRCI is greater than 1 and a city as a regional external advantage (REA) city if its RRCI is less than 1.

**Fig. 7:**
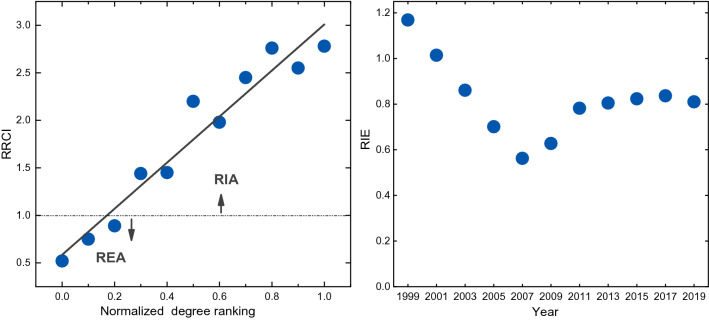
The relation between topology and regional interactions. **a** The relation between the degree (normalized rankings in regions) and the RRCI. **b** The ratio of the number of RIA cities to that of REA cities, referred to as RIE

We utilize both the RRCI and degree ranking to assess a city’s regional role in the dynamics of network topology. As shown in Fig. 7a, the RRCI shows a positive correlation with the degree ranking, meaning that cities with higher degree within region tend to expand their own external connections. High-degree cities are generally planned core cities, which are not only bellwethers of regional development, but also windows to the outside regions. We further show the ratio of the number of RIA cities to the number of REA cities in Fig. 7b. The value initially decreases linearly, and then increases and gradually reaches a plateau.

**Fig. 8:**
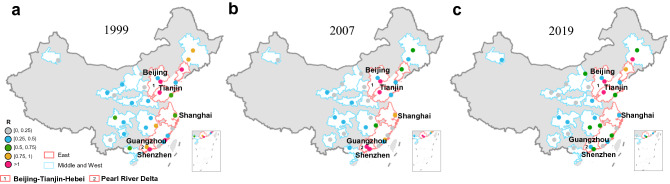
The spatial distribution of RRCI of core cities. The spatial distribution in **a** 1999, **b** 2007, and **c** 2019. The division between the east, west, and central regions is based on the Nation Bureau of Statistics of China (NBSC) [50]

To better demonstrate the regional dynamics of CRN over time, we plot the spatial distribution of RRCI for 1999, 2007, and 2019 in Fig. 8a, b, c, respectively, where nodes are marked with different colors according to their levels of RRCI. For simplification, we take only the core cities, such as municipalities or provincial capitals, into consideration. Spatially, cities with a low RRCI are mostly distributed in central and western China. In eastern China, this index varies widely across different regions. For the Beijing–Tianjin–Hebei region, the two core cities, Beijing and Tianjin, maintain a stable level of RRCI. Beijing is identified as an REA city with low RRCI, and Tianjin as an RIA city with high RRCI. Compared with the Beijing–Tianjin–Hebei region, Pearl River Delta behaves quite differently in the RRCI evolution. In 1999, Guangzhou and Shenzhen had the advantage in outer/inner interactions, respectively. With the increasing train flow within this region, Guangzhou strengthened its internal transport advantage and gradually converted into an RIA city with improved RRCI. Shenzhen continued to maintain the status of an RIA city. Subsequently, long-distance high-speed railway lines were gradually launched into service, including Wuhan–Guangzhou high-speed railway in 2009, Beijing–Guangzhou high-speed railway in 2012, and Guiyang–Guangzhou high-speed railway in 2014. The external railway channels of Pearl River Delta were expanded. As a consequence, both Guangzhou and Shenzhen transformed from REA cities into RIA cities. We further analyze the ratio of RIA/REA cities (referred to as RIE) in the Beijing–Tian–Hebei region and Pearl River Delta (see Table 4). It is easy to see that this ratio in Beijing–Tianjin–Hebei region is much higher than that in Pearl River Delta. Most cities in the Beijing–Tianjin–Hebei region focus on internal interactions with nearby cities, while a small number of cities show a relatively strong regional bridging effect. In contrast to the Beijing–Tianjin–Hebei region, Pearl River Delta has more REA cities.

**Table 4:** Two case examples

	1999	2001	2003	2005	2007	2009	2011	2013	2015	2017	2019
Beijing–Tianjin–Hebei	6	3	5	3	7	2	5	2	2.5	4.5	2.67
Pearl River Delta	1.33	1.33	0.67	1.33	1.5	0.75	1	0.5	0.6	0.4	0.4

The ratio of the number of RIA cities to that of REA cities (RIE) for the Beijing–Tianjin–Hebei region and Pearl River Delta in different years

## Conclusion

This paper has analyzed the evolution of CRN over the period 1999-2019 in respect of factors including topological structure, train flow, and train travel distance. By reviewing the development of China’s railway system, we found that both the railway length and passenger traffic demonstrate a continuous increase at a higher rate. Subsequently, based on complex networks theory, we found that CRN has become more densely connected and heterogeneous with increasingly prominent small-world properties over time. The combined analysis of the train flow and train travel distance shows a two-stage evolutionary process, with the turning point in 2007 when high-speed trains started to be put into operation. Additionally, the spatial distribution of high-flow edges exhibited an obvious change. The edges with large train flow gradually infiltrated into large city pairs and then shifted towards the connections between large cities and their nearby small cities. Finally, we propose an RRCI, based on regional internal/external train flow, to identify RIA and REA cities. Cities with high degrees are consistently found to have an advantage in connecting with cities outside the region and are identified as REA cities, whereas cities with low degrees always exhibit strong internal interactions and are identified as RIA cities.

Our analytical approaches and results may contribute to future decision-making and decision evaluation. Importantly, following the proposed framework, the comprehensive impact resulting from the railway planning implementation can be adequately assessed through long-term experimental validation. Based on the evolution results, decision-makers may clearly identify the fast/slow-growing nodes/edges/areas and re-evaluate the effectiveness of previous decisions. Furthermore, due to the applicability of complex network theory for different transportation modes, the proposed approach can also be applied to explore the network evolution of urban rail systems and help to identify spatiotemporal characteristics.

With the continued expansion of intercity and urban rail networks, transportation network features may become more complex. Given the unique characteristics of different rail networks, our future research will focus on the integrated point-to-point travel process based on multilayer complex network theory. We also suggest that additional indicators such as network reliability, economic conditions, and geographical features should be considered in measuring the importance of network facilities for future research.

## Appendix A: Acronyms

**Table:**

Acronyms	Description
CRN	China’s railway network
IRN	Indian railway network
JRN	Japanese railway network
HSR	High-speed railway
RRCI	Regional railway connection index
RIA	Regional internal advantage
REA	Regional external advantage
RIE	Ratio of number of RIA cities to that of REA cities
